# The clinical value of high fluorescent lymphocytes and smudge cells in the diagnosis of infectious mononucleosis

**DOI:** 10.1002/jcla.23965

**Published:** 2021-08-17

**Authors:** Tao Huang, Shuo Yang, Yufeng He, Qiang Li, Liyan Cui

**Affiliations:** ^1^ Department of Laboratory Medicine Peking University Third Hospital Beijing China

**Keywords:** early diagnosis, high fluorescent lymphocytes, infectious mononucleosis, reactive lymphocytes, smudge cells

## Abstract

**Background:**

The diagnostic value of high fluorescent lymphocytes (HFLC) and smudge cells in diseases like sepsis has been confirmed. In this study, we explore the diagnostic value of HFLC and smudge cells for infectious mononucleosis (IM).

**Methods:**

Sixty‐two IM patients, 67 healthy controls, 84 patients with upper respiratory tract virus infection, and 35 patients with malignant lymphoid diseases were enrolled. The complete blood counts and leukocyte differential counts are tested, and the smudge cells were manually counted.

**Results:**

The value of HFLC% and smudge cells of the IM group were significantly higher than those of healthy controls and disease controls (*p *< 0.05), and the HFLC% value of IM patients was positively correlated with the number of reactive lymphocytes (*r* = 0.265). When the cutoff value of HFLC% was 0.4%, and the diagnostic value of IM was high (AUC = 0.995). When the smudge cells >2/100 nucleated cells, it can show better (AUC = 1.000). When the cutoff value of the HFLC% was 1.2%, it can effectively distinguish IM patients from upper respiratory tract virus infection patients (AUC = 0.934); when smudge cells >16/100 nucleated cells, it also has high differential diagnosis value (AUC = 0.913). In addition, the AUC of the combination HFLC% and smudge cells for the differential diagnosis can be increased to 0.968. The performance value of single HFLC% (AUC = 0.942) for distinguishing IM from malignant lymphoid diseases was better than smudge cells and combine index with the cutoff value of 0.4%.

**Conclusion:**

HFLC% and smudge cells can be used as effective indicators in the early diagnosis and differential diagnosis of IM.

## Graphical abstract

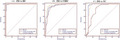

## 1 INTRODUCTION

Infectious mononucleosis (IM) is a clinical disease characterized by fever, angina, and lymphadenopathy. It was first proposed by Sprunt and Evans^1^ to describe similar acute infections. The disease is accompanied by a syndrome of atypical large lymphocytes in the peripheral blood. This atypical lymphocyte is also called Downey cell.^2^ IM is most common in adolescents and young people, and often occurs in late autumn to early spring. It may be caused by a variety of pathogens, and Epstein‐Barr virus (EBV) infection accounted for the vast majority of patients. It can be spread through droplets, blood transfusion etc., and humans are generally susceptible. Therefore, IM is also defined as an acutely proliferative self‐limiting infectious disease of the mononuclear‐macrophage system caused by the EBV. Although the related clinical symptoms of IM such as fever, fatigue, lymph nodes, and hepatosplenomegaly will gradually disappear within a few months, a small number of patients will still experience splenic rupture, chronic active EBV, hemophagocytic syndrome, and other serious and long‐lasting complications.^3^ At present, there is no specific treatment method for IM, and there is no approved EBV vaccine. Therefore, early diagnosis of IM, timely medical intervention, to prevent the aggravation of the disease is of great significance.

Clinically, the diagnosis of IM is mainly based on the comprehensive consideration of clinical symptoms, hematology, and serology, and the following criteria are mostly used: ① fever, angina, lymphadenopathy, hepatosplenomegaly, and skin rash; ② increased proportion of lymphocytes, and reactive lymphocytes>10%; ③ heterophilic agglutination test is positive; ④ anti‐viral capsid antigen (VCA) IgM antibody is positive; and ⑤the syndrome can be caused by other viruses (cytomegalic virus, etc.) and some bacteria. Atypical lymphocytes appear, but the heterophilic agglutination test is negative. When the patient has three symptoms in ①, meets any two of ②③④, and excludes ⑤, it can be diagnosed as IM. In particular, IM patients who are negative for heterophilic antibodies due to other viral agents are also considered. However, these diagnostic standards have their shortcomings: the clinical manifestations described in ① can appear in a variety of diseases, and the indication of IM is not clear, and it is easy to be misdiagnosed as upper respiratory tract infections and other diseases; and the morphology of reactive lymphocytes is diverse, leading to large differences in judgment among different technicians.^4^ The formation of smudge cells leads to serious cell damage and affects the accuracy of manual leukocyte classification. The accuracy of heterophilic agglutination to diagnose IM was 71%–90%; however, the test has a false‐negative rate of 25% in the first week of illness,^5^ and it is a non‐specific test. The high cost of serological antibody testing, long testing time, and short duration of IgM antibodies is prone to miss diagnosis. Therefore, it is very valuable to find novel biomarkers with higher diagnostic efficiency for the diagnosis of IM. In our clinical work, we found that the HFLC% value and the number of smudge cells in patients diagnosed with IM have an increasing trend. The aim of this study was to evaluate the efficiency of HFLC% and smudge cells in the peripheral blood as diagnostic biomarkers of IM.

## 2 MATERIALS AND METHODS

### 2.1 Study population

We conducted a prospective observational study in Peking University Third Hospital from August 2020 to February 2021. IM patients were enrolled with the following criteria: patients who met clinically relevant diagnostic criteria mentioned above and were diagnosed with IM. The exclusion criteria are as follows: ① patients with elevated peripheral leukocytes and a significant increase in neutrophils suggesting bacterial infection and exclude patients infected with cytomegalovirus and mycoplasma pneumonia; ② patients with malignant solid tumors or immune insufficiency; ③ patients with other systemic diseases; and ④patients who had taken cytotoxic drugs or immunosuppressants in the first 3 months of enrollment. According to strict exclusion criteria, a total of 62 patients with IM were enrolled in this study finally, and 84 cases diagnosed as upper respiratory tract infection (viral infections other than EBV infection) were included as upper respiratory tract infection control group (URIC), and 35 patients diagnosed with malignant lymphoid diseases (including chronic B lymphocytic leukemia, lymphoma, diffuse large B‐cell lymphoma, etc.) were served as tumor control (TC). Meanwhile, 67 healthy individuals were recruited as healthy controls (HC). The present study was approved by the ethics committee of Peking University Third Hospital. Written informed consents were obtained from each participant at the time of enrollment.

### 2.2 Sample and data collection

EDTA anticoagulated blood samples (3 ml) were obtained from all the participants in a standard procedure of venous blood collection. Complete blood counts including red blood cells (RBC), hemoglobin (HGB), white blood cells (WBC), and leukocyte differential count, platelet (PLT), and HFLC percentage (HFLC%) were tested by Sysmex XN 9000 (Sysmex Corporation Kobe) within 2 h. Peripheral blood smears were prepared by Sysmex SP‐10 (Sysmex Corporation Kobe) automatic staining machine, the pushing angle was set to 27°, and the speed was set to 110 mm/s. Manual leukocyte classification and smudge cells were counted by Sysmex DI‐60 (Sysmex Corporation Kobe). A total of 200 nucleated cells were read on each slide. The number of smudge cells seen per 100 nucleated cells was counted, and the blood smears were observed by two experienced technicians under an optical microscope.

### 2.3 Statistical analysis

All data analysis was performed using SPSS 24.0 (SPSS, Inc.) and MedCalc 19.6 (MedCalc Software). Continuous variables with non‐normal distribution were shown as median [interquartile range (IQR)], which used non‐parametric Mann–Whitney *U* test to compare between groups. The data with normal distribution were represented as mean ± standard deviation ($\bar{x}$±SD), and comparison between groups was performed by *t* test. The categorical variables were expressed as percentages (%) and compared by chi‐square test. *p *< 0.05 was considered statistically significant. Spearman's test was used to study the correlation between the HFLC% and the number of reactive lymphocytes in the IM group. The clinical performances of different indicators were assessed by receiver operating characteristics (ROC) curve analysis, the area under the curve (AUC), cutoff values, specificity, and sensitivity were calculated.

## 3 RESULTS

### 3.1 Characteristics of the study population

Basal characteristics and complete blood counts of the study population are summarized in Table 1. There was no significant difference in gender in the four groups, while the patients of IM were significantly younger than that of the control groups. This is consistent with the fact that the prevalence of IM is highest among 15–24 years old.^6^ The complete blood counts of the four groups were statistically analyzed. RBC and HGB in IM group were higher than those in the TC group (*p *< 0.05), but there was no significant difference compared with HC and URIC groups (*p *> 0.05). WBC and PLT in IM patients were significantly higher than those in HC and URIC group (*p *< 0.05); however, there was no significant difference compared with those in TC group (*p *> 0.05). Interestingly, HFLC% was significantly higher in IM patients than in the other three groups (*p *< 0.05).

**TABLE 1:** Basal characteristics and complete blood counts of the study population

Characteristics	IM (*n* = 62)	HC (*n* = 67)	URIC (*n* = 84)	TC (*n* = 35)
Male/Female (*n*, %)	40/22 (64.5%)	37/30 (55.2%)	45/39 (53.6%)	22/13 (62.9%)
Age (years)	16.5 (8–26.5)^a^, ^b^, ^c^	41 (32–53)	29 (29–36.8)	59 (49–68)
RBC(×10^12^/L)	4.71 ± 0.47^c^	4.53 ± 0.26	4.74 ± 0.50	4.09 ± 0.84
HGB(g/L)	134.7 ± 14.6^c^	137.0 ± 6.5	141.8 ± 17.5	122.3 ± 24.6
WBC(×10^9^/L)	10.37 (8.54–12.67)^a^, ^b^	6.07 (5.08–7.60)	8.10 (6.40–9.89)	9.21 (6.10–31.48)
PLT(×10^9^/L)	194.8 ± 58.3^a^, ^b^	246.5 ± 41.6	258.8 ± 59.4	179.0 ± 59.5
HFLC (%)	2.4 (1.7–3.6)^a^, ^b^, ^c^	0.0 (0.0–0.2)	0.4 (0.2–0.7)	0.1 (0.0–0.2)

^a^ Expressed that the difference between the IM group and the HC group is statistically significant (*p* < 0.05).

^b^ Expressed that the difference between the IM group and the URIC group is statistically significant (*p* < 0.05).

^c^ Expressed that the difference between the IM group and the TC group is statistically significant (*p* < 0.05).

### 3.2 Comparison of the number of smudge cells among the IM group, HC group, URIC group, and TC group

**FIGURE 1:**
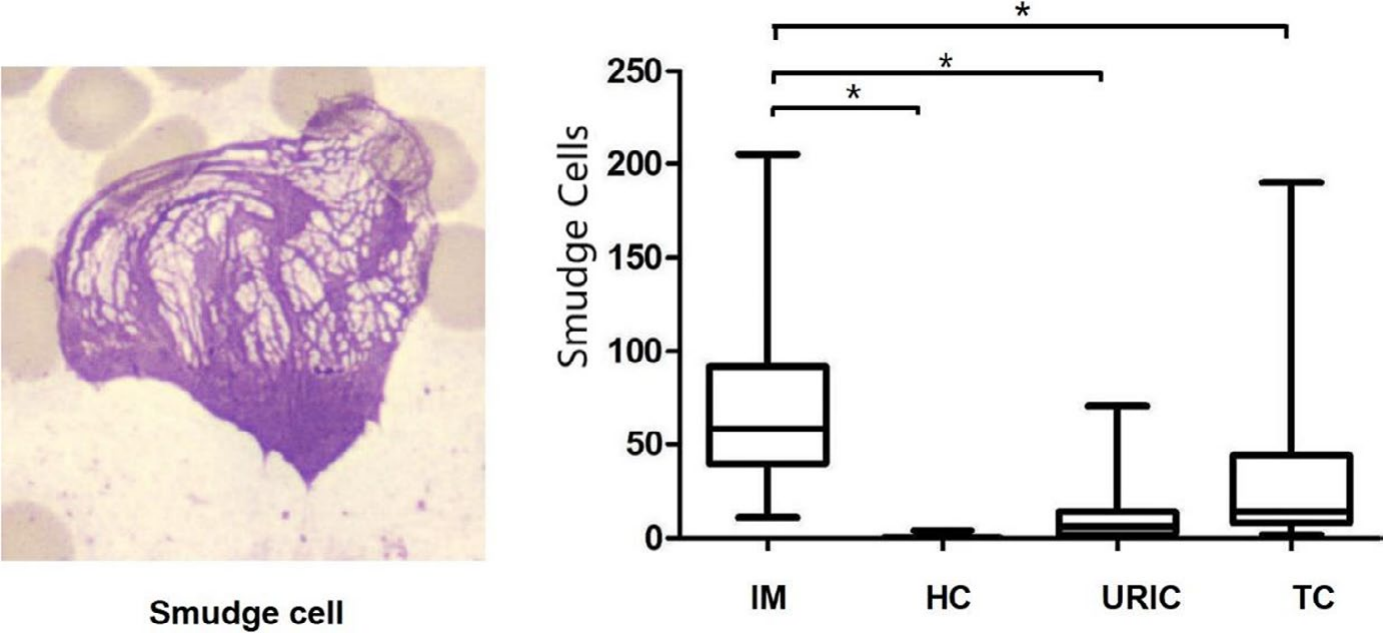
Comparison of the number of smudge cells among the IM group, HC group, URIC group, and TC group; “*”Indicating *p *< 0.05

The number of smudge cells in the four groups of study population was compared and analyzed (Figure 1). The number of smudge cells in the IM group was 29 (20–46) per 100 nucleated cells, which was significantly higher than the HC group [0.0 (0.0–0.0)], URIC [6.0 (2.0–14.0)] group, and TC group [14.0 (8.0–44.0)] (*p *< 0.05).

### 3.3 Comparison of leukocyte differentiation between automatic blood analyzer and manual leukocyte classification

**FIGURE 2:**
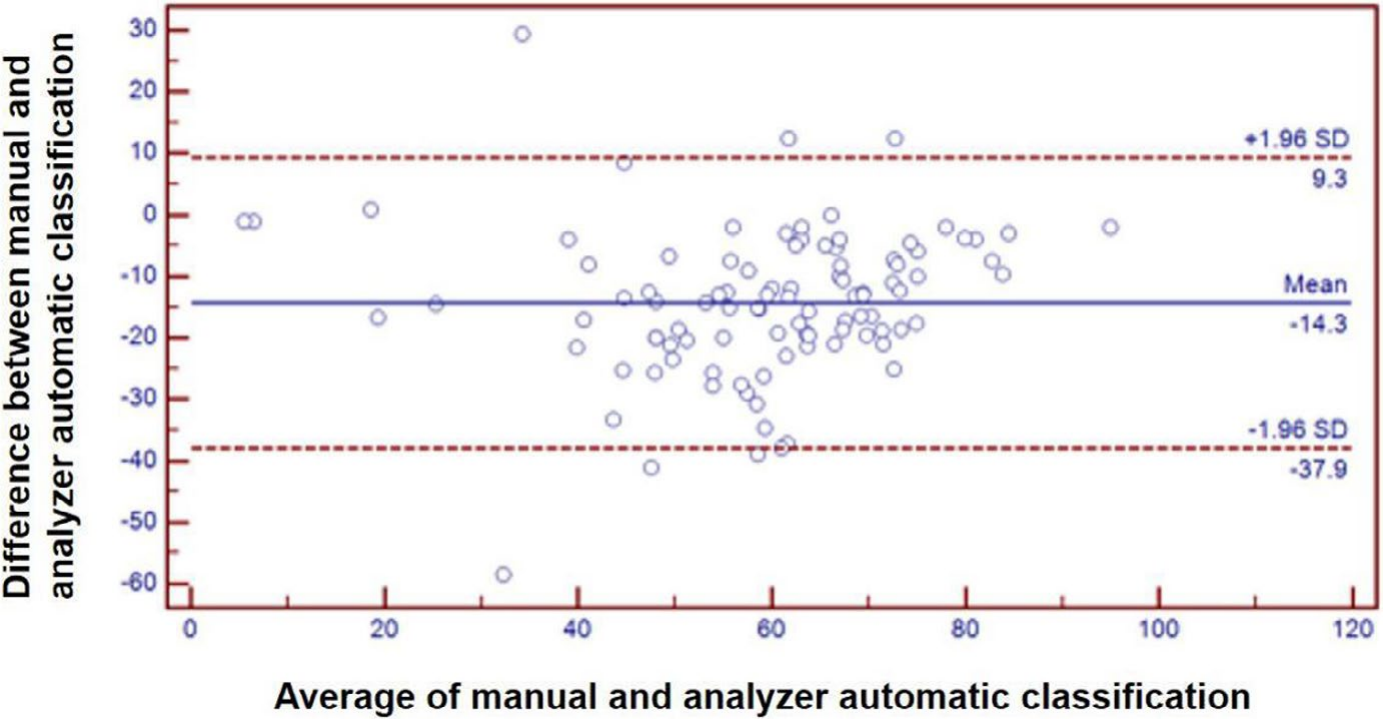
Bland‐Altman deviation diagram of manual classification and analyzer automatic classification results

**FIGURE 3:**
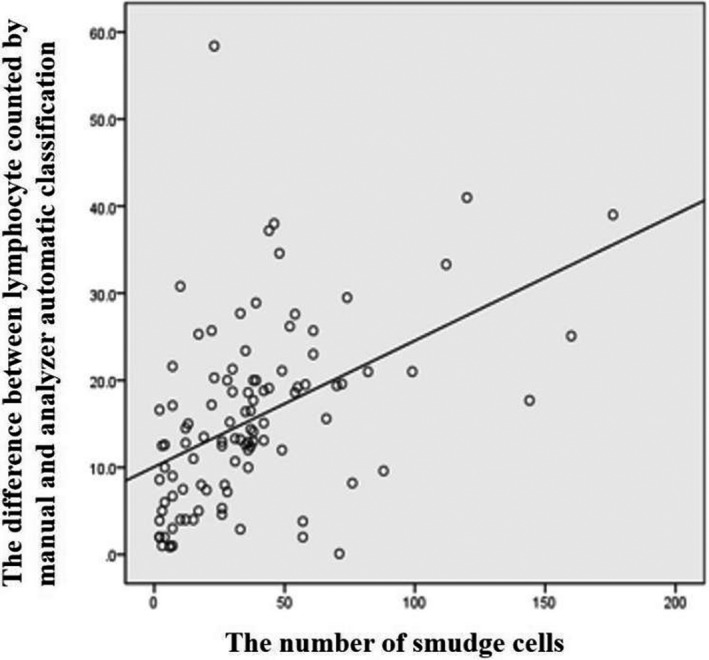
Correlation analysis between the number of smudge cells and the difference in lymphocyte counted by manual and analyzer automatic classification

**FIGURE 4:**
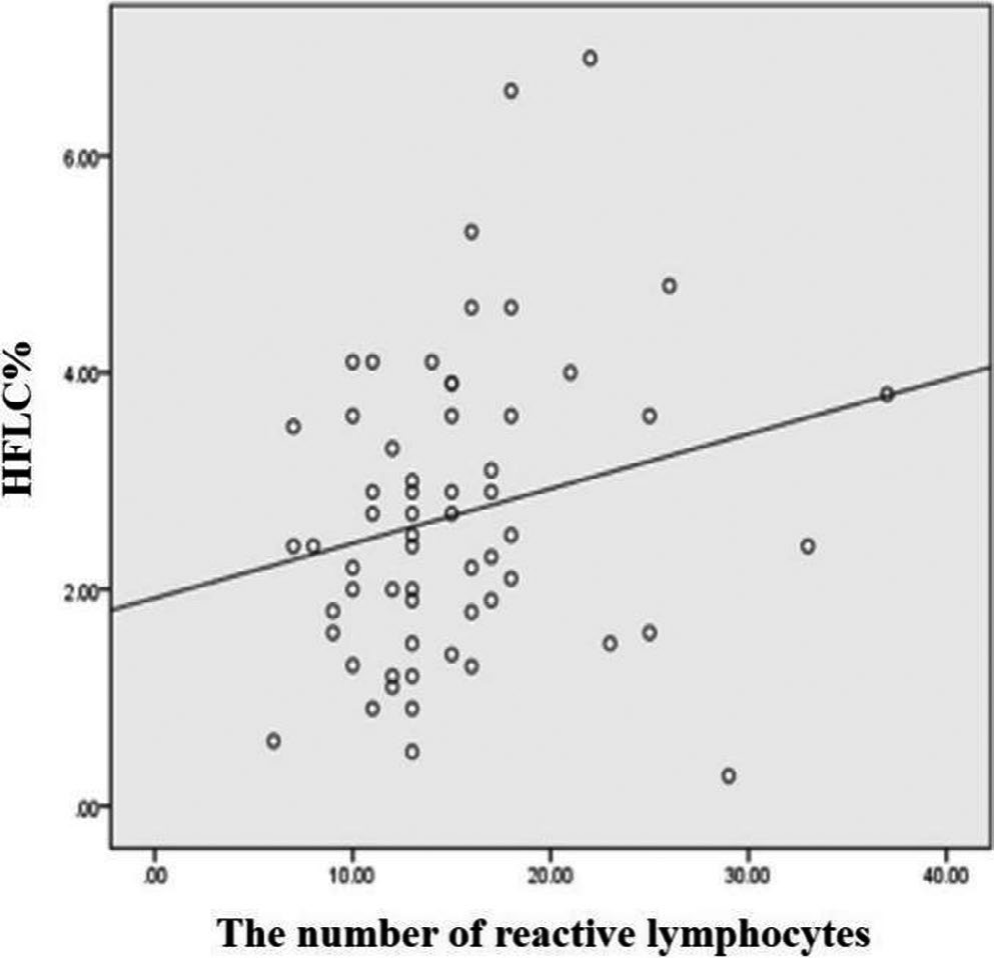
Spearman correlation analysis of HFLC% value and the number of reactive lymphocytes counted by manual classification

We found that there was a significant difference between the percentage of lymphocytes classified by automatic blood analyzer and manual classification (*p *< 0.05). The Bland‐Altman deviation diagram of manual classification and analyzer automatic classification result are shown in Figure 2. The number of lymphocytes counted by manual classification was mostly lower than automatic analyzer. The difference between the number of lymphocytes classified by manual method and by analyzer was positively correlated with the number of smudge cells (*R* ^2^ = 0.213, *p* < 0.05). It suggested that as the number of smudge cells increased, the difference between the percentage of lymphocytes counted by analyzer and manual classification increased (Figure 3). Spearman correlation analysis of HFLC% value and the number of reactive lymphocytes counted by manual classification in the IM group showed that they were positively correlated (*r* = 0.265, *p *< 0.05) (Figure 4).

### 3.4 The diagnostic value of HFLC% and the number of smudge cells for IM

**FIGURE 5:**
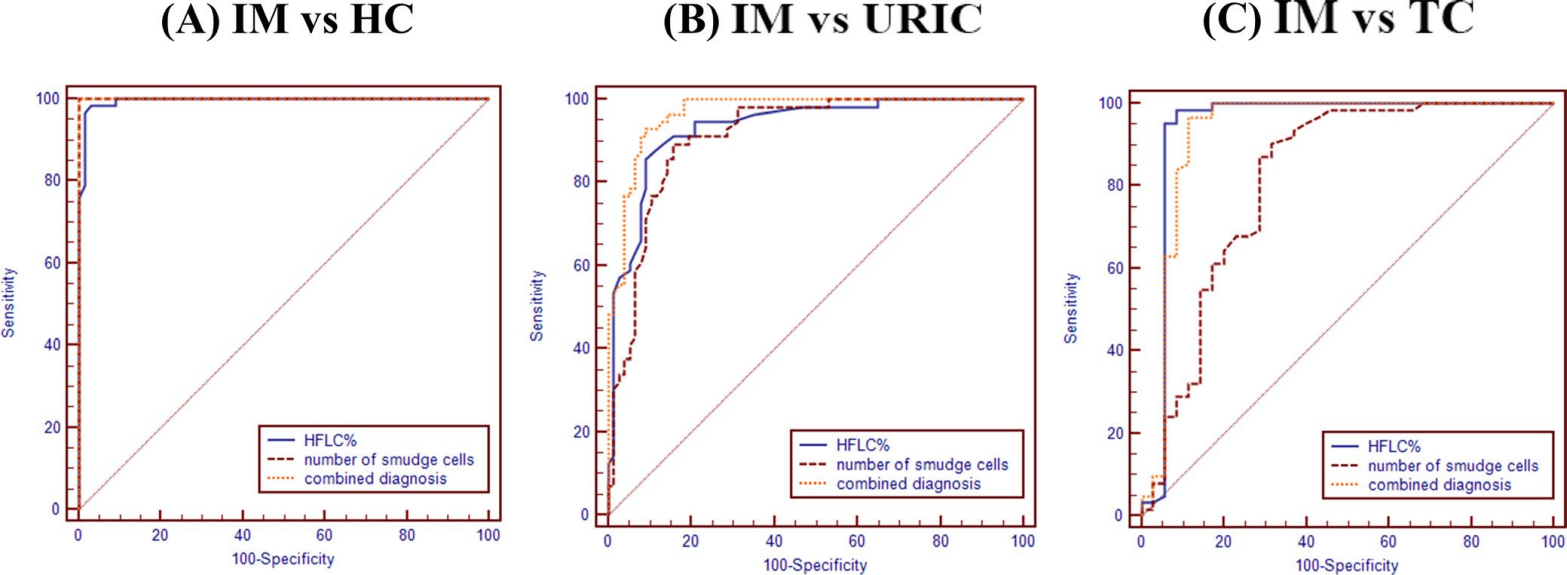
ROC curves of HFLC% and number of smudge cells to diagnose and distinguish IM

The level of HFLC% and the number of smudge cells was significantly higher in patients with IM compared with healthy controls (*p *< 0.05). The AUC (95% CI), specificity, sensitivity, and optimal cutoff values of HFLC%, and smudge cells were shown in Table 2. Both indicators showed good diagnostic value, and the AUC was 0.995(95% CI 0.963–1.000) and 1.000 (95% CI 0.972–1.000), respectively (*p *> 0.05). When the cutoff value of HFLC% was 0.4%, it demonstrated a high sensitivity of 98.39% and a high specificity of 97.01%. The sensitivity and specificity of smudge cells with the cutoff value of 2 per 100 nucleated cells were 100% (Figure 5A).

**TABLE 2:** Diagnostic performance of HFLC% and the number of smudge cells for IM

Biomarker	Cutoff value	Sensitivity	Specificity	AUC	95% CI
HFLC%	0.4%	98.39%	97.01%	0.995	0.963–1.000
Smudge cells (n/100 nucleated cells)	2	100%	Ā%	1.000	0.972–1.000
Combined diagnosis	/	100%	100%	1.000	0.972–1.000

### 3.5 The value of HFLC% and number of smudge cells in differentiating IM from upper respiratory tract virus infection

The optimal HFLC% was >1.2% with a sensitivity of 85.71%, specificity of 90.91% for differentiating IM from upper respiratory tract virus infection, and its AUC was 0.934 (95% CI 0.877–0.970). When the number of smudge cells in the peripheral blood smear was more than 16 per 100 nucleated cells, the differential diagnostic sensitivity of IM was 89.29%, the specificity was 84.42%, and AUC was 0.913 (95% CI 0.852–0.955). Combined detection of HFLC% and smudge cells can significantly increase the AUC to 0.968 (95% CI 0.922–0.991) (*p* < 0.05) (Table 3, Figure 5B).

**TABLE 3:** The performance of HFLC% and number of smudge cells in differentiating IM from upper respiratory tract virus infection

Biomarker	Cutoff value	Sensitivity	Specificity	AUC	95% CI
HFLC%	1.2%	85.71%	90.91%	0.934	0.877–0.970
Smudge cells (n/100 nucleated cells)	16	89.29%	84.42%	0.913	0.852–0.955
Combined diagnosis	/	92.86%	90.91%	0.968	0.922–0.991

### 3.6 The value of HFLC% and the number of smudge cells in differentiating IM from malignant lymphoid diseases

Compared with smudge cells, the value of HFLC% in differentiating IM from malignant lymphoid diseases was much better (*p *< 0.05). Moreover, combined detection of these two indicators could not further improve the performance (Table 4). When cutoff value of HFLC% was 0.4%, the differential diagnosis value of IM and malignant lymphoid diseases was high with a sensitivity of 98.39%, a specificity of 91.43%, and an AUC of 0.942 (95% CI 0.875~0.979). However, when the number of smudge cells in the peripheral blood smear was more than 15 per 100 nucleated cells, the sensitivity of the differential diagnosis of IM was 90.32%, with a low specificity of 68.57%, and an AUC of 0.817 (95% CI 0.725~0.888) (Figure 5C).

**TABLE 4:** The performance of HFLC% and the number of smudge cells in differentiating IM from malignant lymphoid diseases

Biomarker	Cutoff value	Sensitivity	Specificity	AUC	95% CI
HFLC%	0.4%	98.39%	91.43%	0.942	0.875–0.979
Smudge cells (n/100 nucleated cells)	15	90.32%	68.57%	0.817	0.725–0.888
Combined diagnosis	/	96.77%	88.57%	0.930	0.860–0.972

## 4 DISCUSSION

Most people are infected with EBV in childhood and then act as asymptomatic patients of the B lymphatic system. When the balance between EBV and the host is disrupted by some unknown interventional conditions, the virus can exert its pathogenic potential, which may develop clinical manifestations of IM.^7^ After EBV infection, viral replication can first be detected in the oral cavity, and it can infect a variety types of cells such as tonsil epithelial cells and B cells. EBV can be replicated in B cells, activate B lymphocytes transforming their surface antigens and morphology, stimulate the body to produce immune response, produce EBV‐specific cytotoxic T lymphocytes, and target against EBV. Infected B lymphocytes can play an anti‐viral effect and also cause a series of clinical symptoms such as fever and hepatosplenomegaly. Due to the limitations of current diagnostic criteria for IM, it is of great significance to find reliable indicators for the early diagnosis of IM.

**FIGURE 6:**
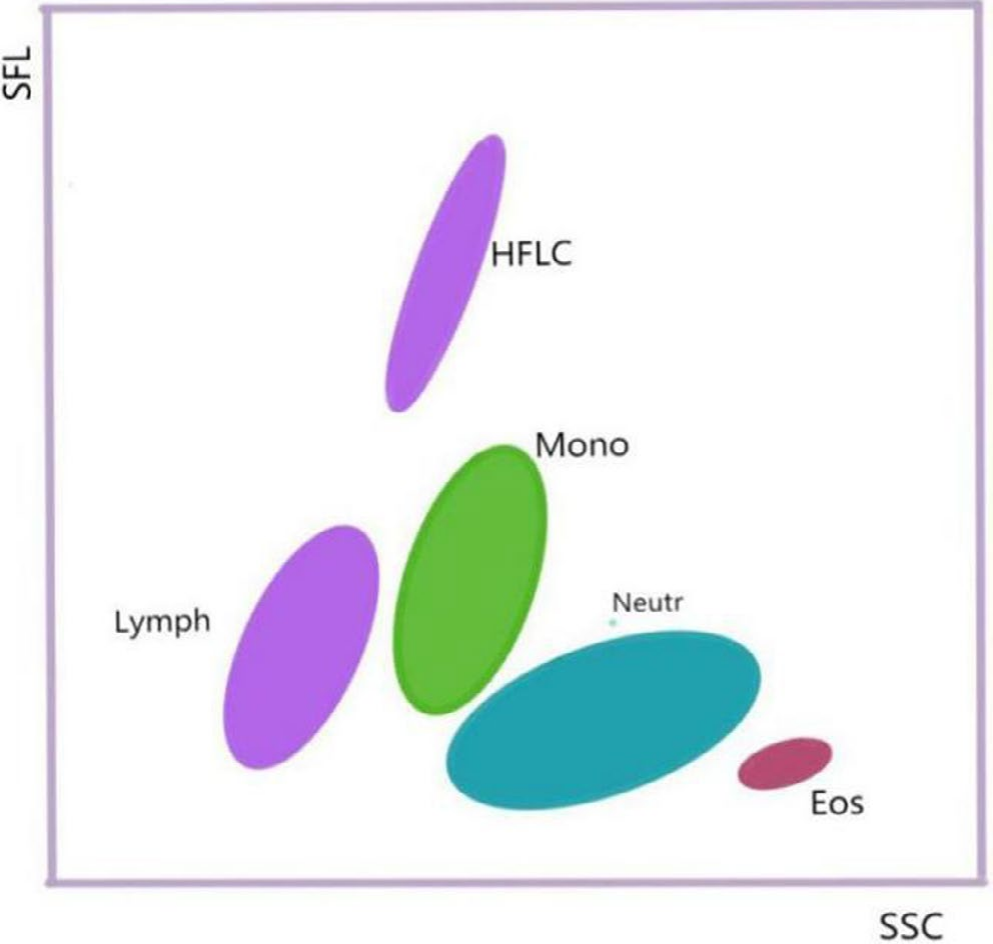
Scatter diagram of leukocyte classification in the WDF channel of the Sysmex automatic hematology analyzer. Eos, Eosinophils; HFLC, high fluorescent lymphocytes; Lymph, lymphocytes; Mono, monocytes; Neutr: neutrophils+ basophil; SFL, side fluorescence; SSC, side scatter

The new generation of Sysmex hematology analyzer combines flow cytometry and nucleic acid fluorescence staining technology to classify and count leukocytes.^8^ HFLC count is one of the hematology research markers analyzed using this automated instrument, revealing a population of lymphocytes with high fluorescence intensity (Figure 6). Nucleic acid substances are significantly increased as lymphocytes activated after antigen stimulation, and HFLC can bind more nucleic acid fluorescent dyes to increase the fluorescence signal, so this cell population can be distinguished by higher fluorescence signal intensity compared with monocytes and normal lymphocytes in the scatter diagram of leukocytes classification.^9^

Previous studies^8^, ^10^ have shown that HFLC can identify and quantify activated B lymphocytes and peripheral plasma cells with high accuracy and reliability, thereby it provided tools for assisting diagnosis and monitoring the course of patients suspected of infection. Many studies have confirmed that HFLC can be used as a new immunological indicator to guide clinical diagnosis. Chaicharoen Tantanate et al. found that there was no significant difference in HFLC among patients with immune system diseases, malignant tumors, and other diseases, while the HFLC in infectious disease group was significantly higher than healthy individuals and other disease groups (*p* < 0.001). In the infectious disease group, patients with dengue fever accounted for the largest proportion (155/229). The analysis showed that the counts of HFLC in patients infected with dengue fever were significantly higher than that of patients infected with other pathogens (*p* < 0.001), indicating that HFLC has clinical value in the diagnosis of patients infected with dengue fever.^9^ Arneth BM et al. found an early elevation of HFLC in a patient who developed from a urinary tract infection to uremia. They conducted a retrospective study HFLC on 38 patients in intensive care units (ICU) to further research. The results showed that in the group of non‐infection patients, the HFLC value was very low, the reference range was less than 0.2%, while in the local infection group, the value increased slightly, and it increased significantly in the patients with sepsis, suggesting that HFLC has auxiliary diagnostic value in sepsis.^11^

Our study confirmed that HFLC% has a good clinical diagnostic value in IM patients. In clinical practice, the most common diseases with lymphocyte increase are upper respiratory tract virus infection and malignant lymphoid diseases. Therefore, these two diseases, which are similar to IM in clinical presentation and laboratory examination, were used as control groups in this study to evaluate the value of the novel indicators in the diagnosis and differential diagnosis of IM. HFLC% value of the IM patients was significantly higher than that of the healthy individuals and the disease control groups (*p* < 0.05). When the cutoff value was 0.4%, IM patients can be identified from healthy controls with a high sensitivity of 98.39%, a high specificity of 97.01%, and an AUC of 0.995 (95% CI 0.963–1.000). In addition, with this cutoff value, HFLC% can effectively distinguish IM from malignant lymphoid diseases, whose AUC was 0.942 (95% CI 0.875–0.979), sensitivity was 98.39%, and specificity was 91.43%. For patients with upper respiratory tract virus infection, HFLC% also showed excellent differential diagnosis value. When its cutoff value was 1.2%, the sensitivity and specificity of differential diagnosis were 85.71% and 90.91% respectively, and the AUC was as high as 0.934 (95% CI 0.877–0.970), it can sensitively and specifically distinguish patients with IM and upper respiratory tract infections. In addition, we found that HFLC% was positively correlated with the number of reactive lymphocytes (*r* = 0.265, *p *< 0.05), but there was no significant correlation with the number of normal lymphocytes (*p *> 0.05).

In addition to HFLC%, we also found that the number of smudge cells had similar performance for the diagnosis of IM. Smudge cells are white blood cells that ruptured or damaged during the preparation of the peripheral blood smear. They can be expressed as naked nuclei separated from the ruptured cells. This naked nucleus is different from the well‐preserved nucleus without cytoplasm. The contents are often arranged in a thick network of lines, so they are also called basket cells.^12^ The smudge cells are considered to be artificial during the preparation of peripheral blood smears, which can reflect the fragility of lymphocytes. The smear cells have obvious morphological characteristics, which are easier to identify compared with reactive lymphocytes, and have better comparability among different technicians. Smudge cells as a prognostic factor of chronic lymphocytic leukemia have been fully studied.^13^ In viral infections, this indicator also shows good specificity.^12^ In this study, we found that the presence of a large number of smeared cells seriously affected the accuracy of manual classification of white blood cells. The greater the number of smeared cells, the much more difference between automatic and manual classification of lymphocytes. Interestingly, the results of this study showed that smudge cells can represent extremely high diagnostic efficiency in distinguishing IM patients from healthy controls. When the cutoff value was 2 per 100 nucleated cells, the AUC was 1.000 (95% CI 0.972–1.000). For patients with upper respiratory tract virus infection with clinical symptoms similar to IM, smudge cells also showed good differential diagnostic value. The AUC was 0.913 (95% CI 0.852–0.955) with the cutoff value of 16 per 100 nucleated cells. However, when distinguishing from malignant lymphoid diseases, its performance was not better than HFLC%. Compared with a single indicator, the combined detection of HFLC and smear cells had better ability to distinguish IM and upper respiratory tract infections, and the AUC was increased to 0.968 (95% CI 0.922–0.991) (*p *< 0.05).

There are some limitations in this study. First of all, after the SARS‐CoV‐2 epidemic broke out, people became more aware of virus protection and began to wear masks. As a result, the number of patients with IM who went to the hospital decreased compared with previous years, which resulted in a limited number of samples in this study. To determine the disease‐specific cutoff value and reference range, larger and more diverse sample data are necessary to be included. In addition, the influence of disease progression, drug use, lifestyle, age, and other factors on the HFLC% level of patients with IM is still uncertain, and further research is needed.

## 5 CONCLUSION

HFLC% can be used for auxiliary diagnosis of IM, thereby saving time and reducing cost of smear and manual classification. HFLC% assists the diagnosis of IM with the following advantages. First, this indicator has high specificity and sensitivity for the diagnosis of IM, which can effectively avoid missed diagnosed. Second, HFLC%, as one of the blood routine indicators, can be obtained directly from the automatic blood analyzer. It does not increase additional economic burdens and manual operations. It has better adaptability and superiority and can prompt clinical diagnosis and treatment earlier. In addition, when the number of smudge cells in the peripheral blood increases, the doctor should be alert to the possibility of IM.

## Data Availability

The data that support the findings of this study are available on request from the corresponding author. The data are not publicly available due to privacy or ethical restrictions.
